# Screening for Parasitic Infection and Tuberculosis in Immunosuppressed and Pre-Immunosuppressed Patients: An Observational Study

**DOI:** 10.3390/tropicalmed6030170

**Published:** 2021-09-21

**Authors:** Luisa Carnino, Jean-Marc Schwob, Dionysios Neofytos, Maria Lazo-Porras, François Chappuis, Gilles Eperon

**Affiliations:** 1 Division of Tropical and Humanitarian Medicine, Geneva University Hospitals, Rue Gabrielle-Perret-Gentil 6, 1205 Geneva, Switzerland; jean-marc.schwob@hcuge.ch (J.-M.S.); maria.lazo.porras@gmail.com (M.L.-P.); francois.chappuis@hcuge.ch (F.C.); gilles.eperon@hcuge.ch (G.E.); 2 Faculty of Medicine, University of Geneva, Rue Michel-Servet 1, 1205 Geneva, Switzerland; 3 Division of Infectious Diseases, Geneva University Hospitals, Rue Gabrielle-Perret-Gentil 6, 1205 Geneva, Switzerland; dionysios.neofytos@hcuge.ch; 4 CRONICAS Center of Excellence in Chronic Diseases, Universidad Peruana Cayetano Heredia, Lima 15102, Peru

**Keywords:** parasitological screening, latent tuberculosis infection, immunosuppression, *Strongyloides stercoralis*, *Trypanosoma cruzi*, *Echinococcus multilocularis*, *Entamoeba histolytica*, *Leishmania* spp.

## Abstract

Reactivation of latent tuberculosis infection (LTBI) or latent parasitic infection (LPI) during drug-induced immunosuppression can have serious consequences. The Division of tropical and humanitarian medicine of the Geneva University Hospitals runs a specific consultation for parasitic screening of immunosuppressed or pre-immunosuppressed patients. We sought to determine the seroprevalence of LTBI and LPI in such patients and explore its relationship with country of origin or previous travel in a retrospective, single-centre observational study from 2016 to 2019. Demographic data, travel history, ongoing treatments and results of the parasitological (*Strongyloides stercoralis*, *Trypanosoma cruzi*, *Echinococcus multilocularis*, *Entamoeba histolytica* and *Leishmania* spp.) and TB screening were collected to calculate LPI or LTBI prevalence. Risk factors for LTBI and strongyloidiasis were analysed using Poisson regression with robust variance. Among 406 eligible patients, 24/353 (6.8%) had LTBI, 8/368 (2.2%) were positive for *Strongyloides stercoralis* infection, 1/32 (3.1%) was positive for *Entamoeba histolytica* and 1/299 (0.3%) was positive for Leishmaniasis. No cases of *Trypanosoma cruzi* (0/274) or *Echinococcus multilocularis* (0/56) infection were detected. Previous travel to or originating from high-prevalence countries was a risk factor for LTBI (PR = 3.4, CI 95%: 1.4–8.2 and 4.0, CI 95%: 1.8–8.9, respectively). The prevalence of serological Strongyloidiasis in immunosuppressed patients is lower in comparison to those without immunosuppression (PR = 0.1, CI 95%: 0.01–0.8). In conclusion, screening before immunosuppression needs to be individualized, and LTBI and LPI need to be ruled out in patients who originate from or have travelled to high-prevalence countries. The sensitivity of strongyloidiasis serology is reduced following immunosuppression, so an algorithm combining different tests or presumptive treatment should be considered.

## 1. Introduction

The reactivation of latent tuberculosis infection (LTBI) or parasitic infection (LPI) during drug-induced immunosuppression can have serious consequences on patients’ health. The number of patients receiving immunosuppressive therapy has been increasing following the ongoing introduction of new immunosuppressive and immunomodulatory (ISIM) agents for treatment of autoimmune diseases, and solid and hematologic malignancies. It is estimated that between 2.8% and 6% of the American population takes such treatments [1,2]. A higher degree of immunosuppression means higher risk of the acquisition or reactivation of imported parasitic diseases [3]. Currently, there are no clear guidelines on which type of (and if) LPI screening should be performed before starting most ISIM regimens.

The Geneva canton is a multicultural, high-income area in Switzerland with more than 500,000 inhabitants, in which about 2/3 of its documented population has a foreign citizenship and half was born abroad [4,5]. Over 30% of the population comes from the Mediterranean basin, and approximately 10% are equally distributed between sub-Saharan Africa, Latin America and Asia [4]. In addition, it is estimated that over 10,000 undocumented immigrants, mostly from Latin America and Africa, live in the canton [6]. As such, the situation bears similarities to many other urban centres of high-income countries. Since most ISIM drugs are used in the European and North American markets, little attention has been paid to recommendations regarding screening for tropical/imported parasites. However, in Geneva and elsewhere, an increasing proportion of patients on ISIM had previous exposure to such pathogens. At the Division of Tropical and Humanitarian Medicine (Service de Médecine Tropicale et Humanitaire, SMTH) of the Geneva University Hospitals (Hôpitaux Universitaires de Genève, HUG), immunosuppressed or pre-immunosuppressed patients are screened for tuberculosis and a number of parasites (*Strongyloides stercoralis*, *Trypanosoma cruzi*, *Echinococcus multilocularis*, *Entamoeba histolytica*, *Leishmania* spp.), based on expert opinion [7,8,9,10,11]. The aim of this study was to retrospectively assess the prevalence and risk factors for LTBI and LPI of patients treated with ISIM agents.

## 2. Methods

### 2.1. Study Design, Setting and Participants

**Figure 1:**
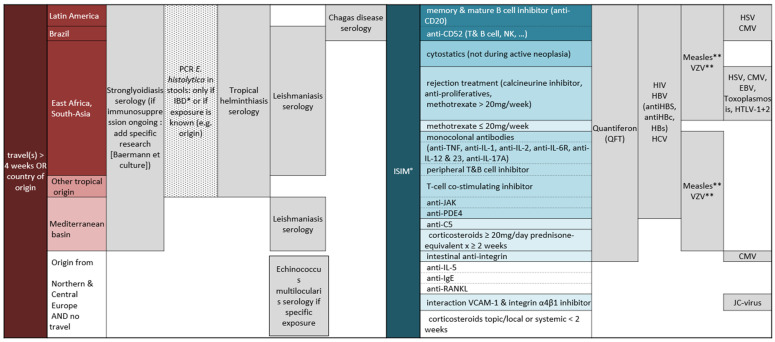
Screening algorithm according to travel exposition and type of immunosuppression ^×^; ^×^ The algorithm was adapted from Eperon et al., RMS 2018, 14: 922–933; * IBD: inflammatory bowel disease; ° ISIM: immunosuppressive and immunomodulatory agents; ** If negative, vaccinate before immunosuppression. QFT: Quantiferon; HIV: Human immunodeficiency virus; HBV: Hepatitis B virus; HCV: Hepatitis C virus; VZV: Varicella-zoster virus; HSV: Herpes simplex virus; CMV: Cytomegalovirus; EBV: Epstein–Barr virus; HTLV-1: human T-cell lymphotropic virus type 1; JC-virus: John Cunningham virus.

This was a retrospective, single-centre, cross-sectional, observational study. The eligible population included patients 18 years old and over who had attended the consultation for immunosuppressed patients at SMTH between December 2016 and December 2019. The main objective of this consultation was to update immunization before or during treatment with one or more ISIM drugs. Furthermore, since 2016, screening for LTBI, viral cosmopolitan infections (HIV, HBV, HCV, CMV, EBV, HSV, data not shown) and LPI screening has been offered (Figure 1). Included in this study were: patients affected by all types of chronic conditions for which ISIM treatment may be prescribed, already treated with ISIM drugs or for whom an impending treatment with one of those drugs, as listed in Supplementary Material Table S1, was planned. Most of the patients had been diagnosed with an immune-mediated inflammatory disease and were referred by different specialists in neurology, dermatology, rheumatology and oncology. The study was approved by the local Ethics Committee (Canton of Geneva, approval No. 2020-01647). Patients whose medical charts documented their refusal to participate in a clinical research study were excluded.

### 2.2. Screening Test Indications

Patients were screened following the algorithm shown in Figure 1. The Quanti-FERON-TB Gold^®^ (QFT, Germantown, MD, USA) test for the diagnosis of latent tuberculosis was recommended according to the type of immunosuppressive treatment. Serological screening for *Strongyloides stercoralis*, *Trypanosoma cruzi*, *Entamoeba histolytica*, *Leishmania* spp. was performed depending on previous exposure in endemic regions (travel or having lived more than 4 weeks in a high-risk country), while screening for *Echinococcus multilocularis* was proposed since July 2019, in case of a specific exposure (e.g., living in a farmhouse, consumption of grown leaf or root vegetable, ingestion of food potentially contaminated by fox feces, owning dogs or cat that roamed outdoors unattended) [12]. Finally, a PCR stool test for amoebiasis was performed according to travel exposition and in patients affected by an inflammatory bowel disease (IBD).

### 2.3. Laboratory Tests

Type of screening test and cut-off values are shown in Table 1. Depending on availability, the tests were performed at HUG, at the Swiss Tropical and Public Health Institute of Basel (Swiss TPH) or at the Institute for Infectious Disease of the University Hospital of Bern. For strongyloidiasis, the serological screening method changed during the time frame of the study: from 2016 to September 2018 an in–house ELISA IgG (*S. ratti* antigen) was used at the Swiss TPH with a cut-off for a positive result at ≥0.7 optical density (OD). From October 2018 onwards, a confirmation test (ELISA IgG Euroimmune^®^, Lübeck, Germany; *S. papillosus* antigen) was added if the in-house screening test result was ≥0.5 OD. The serological result was considered as positive according to the definition used at the time the test was performed.

### 2.4. Data Collection

All data were collected between July 2020 and May 2021, from electronic records of the SMTH consultations, and were managed using REDCap^®^ electronic data capture tools hosted at HUG. Sociodemographic and clinical variables were defined: sex, age, nationality, country of origin, disease for which the immunosuppression was prescribed and a history of travel to geographical regions endemic for the parasites for which screening was proposed: Asia, Africa, Latin America, the Mediterranean basin and the Middle East. The variable travel was treated as a categorical variable: (1) no travel or risk-prone travel less than 4 consecutive weeks, and (2) travel or having lived more than 4 consecutive weeks (including those who were born in endemic countries).

### 2.5. Statistical Analysis

To calculate the required sample size, a small exploratory analysis was performed on a sample of 100 patients who had attended the SMTH consultation. In this sample the prevalence of LTBI was estimated at 8%. Based on this finding, considering an alpha error of 2.5% we obtained a sample size needed of 457 patients. Statistical analysis was performed using StataCorp 2019 (Stata Statistical Software: Release 16, StataCorp LLC, College Station, TX, USA). Descriptive analyses were performed to describe the baseline demographic and clinical characteristics of the study population. Prevalence of LPI or LTBI was estimated by dividing the total number of patients tested positive by the total number of patients screened. For the two most prevalent diseases, LTBI and strongyloidiasis, a bivariate analysis using 2 × 2 tables and chi-square test or Fisher exact were done to explore their association with sociodemographic and clinical characteristics like immunosuppression, travel exposure, country of origin, age and sex. Additionally, risk factors for LTBI and strongyloidiasis were analysed using Poisson regression model with robust variance to estimated prevalence ratios (PR) and their respective 95% confidence interval (95% CI). Only unadjusted models were estimated for strongyloidiasis because of the small number of events, while the model with tuberculosis as an outcome was also adjusted by immunosuppression; that was considered a confounding factor because it reduces the sensitivity of the QFT [13] and can reduce a patient’s travel.

## 3. Results

From a total of 570 patients that attended the immunosuppressed consultation at SMTH, 406 met the study inclusion criteria and were included for further analyses. Excluded patients were those who refused to participate in clinical research study, had no indication to perform a screening test due to lack of exposure or type of immunosuppression and those who refused to perform the tests. A total of 259 (63.7%) patients were females and the median age was 42.2 (range 18–84) years old. Most patients were born in Switzerland, 20.7% were native of a Mediterranean country or the Middle East, while 5%, 4.7% and 3% of patients were native of Latin America, Africa and Asia, respectively. More than half of the patients were immunosuppressed at the time of the QFT (52%) or serological screening (57.6%). Table 2 summarizes the patient baseline demographic and clinical characteristics.

### 3.1. Prevalence of Parasitic and Latent Tuberculosis Infection

The most frequently performed diagnostic test was *Strongyloides stercoralis* serology (368/406, 90.6%), followed by QFT (353/406, 86.9%) and *Leishmaniasis* spp. serology (299/406, 73.6%) (Table 3). The QFT test was positive in 6.7% (24/353) of the patients tested. The seroprevalence of strongyloidiasis was 2.2% (8/368). The improved faecal technique stool test for the direct detection of *Strongyloides stercoralis* larvae performed in 38 patients remained negative in all cases. Latent amoebiasis (1/32, 3.1%) and leishmaniasis (1/299, 0.3%) were diagnosed in one patient each. No cases of *Trypanosoma cruzi* (0/64) or *Echinococcus multilocularis* infection were detected (0/56). During the time frame of the study no patients developed active Tuberculosis or Strongyloides hyperinfection.

### 3.2. Risk Factor for LTBI and Strongyloidiasis

Having travelled > 4 consecutive weeks or having an origin from a high-prevalence country was a risk factor for LTBI after adjustment for immunosuppression (PR = 3.4, 95% CI: 1.4–8.2 and 4.0, 95% CI: 1.8–9.9, respectively). For strongyloidiasis, the prevalence of this condition in those with an immunosuppressed status was 90% less in comparison with those not immunosuppressed (PR = 0.1, 95% CI: 0.01–0.8). Travel exposure and originating from an endemic country was not statistically associated with positive screening test (PR = 1.7, 95% CI: 0.4–7.0, and 3.2, 95% CI: 0.8–13.4, respectively) (Table 4).

## 4. Discussion

To our knowledge this study is one of the first to evaluate the results of a systematically performed combined LTBI and parasitic screening in immunosuppressed patients. We report a prevalence of 6.7% (95% CI 4.6–10) for LTBI and 2.2% (95% CI: 0.9–4.2) for strongyloidiasis in 406 patients with various chronic conditions and ongoing or impending ISIM drug treatment.

Different studies have shown a LTBI prevalence ranging from 3% to 13% in high-income countries, from 10% to 20% in southern and eastern Mediterranean and Latin America and, from 20% to 30% in sub-Saharan Africa, Indian subcontinent, and south-east Asia [14,15]. The prevalence founded in the population studied is consistent to its mixed origins.

Having travelled >4 consecutive weeks or originating from a high-prevalence country increased the prevalence of LTBI in 3.4 and 4.0 times, respectively. The decision to screen for LTBI need to be guided by the type of ISIM treatment prescribed (Figure 1), but a detailed background history and risk-factor assessment (travel or originating from endemic areas) should be part of the anamnesis of the immunosuppressed patient [16].

Variable consensus guidelines recommend routine screening for LTBI before initiation of different types of ISIM [7,17]. In contrast, there are less data on the screening of imported parasitic disease in that context, resulting in lack of standard guidance [18]. The strongyloidiasis seroprevalence found in our study is consistent with what was previously found among renal allograft recipients in Austria (3%), another non-endemic European country [19]. Travel exposure and origin from an endemic country was not associated with a positive screening test, probably due to insufficient power of the study. Considering the lethal risk of hyperinfection syndrome in case of immunosuppression and the availability of a safe treatment simple to administer (oral ivermectin), screening for strongyloidiasis before or during immunosuppression for patients with current or past exposure in an endemic region appears to be justified and should be implemented, even in low-prevalence settings.

It is known that immunosuppression reduces the sensitivity of *Strongyloides stercoralis* serological tests [20], and this likely explains why we found a lower seroprevalence in immunosuppressed than in non-immunosuppressed patients (PR = 0.1). There is therefore the paradox that people with a higher risk of developing a more severe infection are more difficult to diagnose. For this reason, it is advisable to test patients prior to initiation of immunosuppressive treatments, if feasible. Furthermore, the gold standard for the diagnosis of latent strongyloidiasis should include both a serological test and an improved faecal technique stool test. If this dual screening cannot be implemented, a preventive treatment is recommended for patients coming from highly endemic regions before starting a chronic immunosuppressive treatment, i.e., ivermectin 200 mcg/kg once a day for two days [10,21]. This same recommendation is currently being reiterated for patients infected with SARS-CoV-2 coming from high endemic regions who are undergoing corticosteroid therapy [22].

A single case with positive serology for leishmaniasis was detected. Viscerotropic leishmaniasis can reactivate during immunosuppressive treatment, as has been shown for solid-organ transplant recipients and other states of immunosuppression [23]. The prevalence of leishmaniasis is low in Europe (0.02–0.49/100,000, but higher in southern Europe) [24]; therefore, screening for this parasite, at least in central and northern Europeans, seems not justified. Considering the findings of our study, we have changed our screening practices accordingly at the SMTH.

The single case of amoebiasis was a female of Indian origin who had just returned from her home country, and the decision to perform the stool screening test according to existing criteria was justified (Figure 1). No case of *Trypanosoma cruzi* infection (Chagas disease) was detected at our consultation. However, during the time period of the study, two patients from Bolivia with positive *Trypanosoma cruzi* serology were identified before starting their immunosuppressive therapy and were referred to the SMTH consultation. They were not included since the tests had been performed outside our institution. With Geneva being particularly cosmopolitan and having a strong immigration from Bolivia, Chagas disease is well known by the local medical community [25]. As a result, many such immigrants are screened at their first contact with the healthcare system at our institution, perhaps more consistently as compared to centres where the disease is less known. It is very important to raise awareness for Chagas disease, given its high prevalence in Latin America and the increasing movements of populations from this continent around the globe, as the disease can reactivate during immunosuppression with fatal outcomes if not treated [26]. No case of *Echinococcus multilocularis*, an endemic but rare infection in Switzerland (annual incidence 0.15/100,000 [27]) was detected in our study. However, as immunosuppression can increase the incidence and the morbidity of the disease, [28] screening immunosuppressed patient with clear risk factors [12] seems justified. Serology sensitivity is normally above 90% [29] but can be reduced by immunosuppression [28]. Abdominal ultrasound (US) is too expensive and time consuming to be used as a screening test considering the low prevalence of the disease. In our centre we will continue to use serological screening in case of clear exposition and reserve the US in a “case–by-case” management for immunosuppressed patients and for symptomatic patients with non-specific gastrointestinal symptoms or elevation in liver enzyme.

The main limitation of our study was the fact that it was single centre with a relatively small sample size, which restrained the detection of rare infections (e.g., *Enterococcus multilocularis, Leishmania* spp.) and the identification of risk factors for most latent infections. Expanded multicentric studies should be performed to assess the prevalence of these imported parasitic infections and their risk factors in low-prevalence countries, and to design evidence-based and cost-effective strategies for their detection and management. Another possible bias was that in Switzerland, undocumented migrants may have less access to specialized medical services because purchasing private health insurance is mandatory to access the healthcare system and sometime franchises need to be paid out of pocket. The Swiss canton of Geneva has implemented primary care services within the public healthcare system for undocumented migrants that cannot purchase insurance, but probably they still receive less care than the general population due to administrative barriers and fear of denunciation [5]. This could explain the fact that a smaller proportion of our patients were born outside Europe in comparison to the Geneva general population, in association with the fact that the majority of patients were affected by multiple sclerosis, which is more prevalent in Caucasian patients [30].

The changes in diagnostic procedures for strongyloidiasis could have introduced a bias because patients with a doubtful screening test are now undergoing a confirmation test, while before September 2018 they were considered as negative. Immunosuppression is known for reducing the sensitivity of strongyloidiasis serology [20] while it does not affect the result of the Baermann or culture stool tests, and for this reason a dual test (serology and stool) is recommended [11].

In the study population, the implementation of the stool test was irregular, deviating from the algorithm described in Figure 1 due to practical (the need of a second consultation, the requirement that the stool sample be analysed within a 4 h delay) and economic reasons, so the *Strongyloides* prevalence could have been underestimated. On the contrary, even if it represents a worldwide trend, Geneva is a region with a high proportion of immigrants, and the prevalence of the diseases that we found are unlikely to be typical of regions with less international admixture.

## 5. Conclusions

Screening before immunosuppressive therapy needs to be individualized depending on the patient’s exposure to pathogens: strongyloidiasis, amoebiasis and Chagas disease need to be excluded if the patient is native or has travelled for several consecutive weeks to a high-prevalence region. LTBI needs to be ruled out according to ISIM agents and for risk-factor assessment. For strongyloidiasis, in immunosuppressed patients a combined screening approach (serology and stool testing) is indicated. As *E. multilocularis* is endemic in Switzerland, screening patients with specific exposure is probably justified. All these parasitic infections belong to the group of neglected tropical diseases (NTDs), and it is not surprising that they have been so little studied, also in this particular context. Calling for more studies to improve detection and management of these conditions is part of the fight against this neglect.

## Figures and Tables

**Table 1:** Screening test type and cut off.

Pathogen	Test	Cut Off Value
*Mycobacterium tuberculosis*	Quantiferon^®^	Positive ≥ 0.35 IU/mL Indeterminate: blood cells have not responded to a positive control stimulant. Negative < 0.35 IU/ml
*Strongyloides* spp.	In–house ELISA IgG (*S. ratti* antigen) SWISS TPH	Positive > 0.7 OD Doubtful ≥ 0.5 ≤ 0.69 OD Negative < 0.5 OD
	Euroimmune^®^ ELISA IgG (*S. papillosus* antigen) Swiss TPH	Negative < 0.8 Doubtful ≥ 0.8 to < 1.1 Positive ≥ 1.1
	Baermann test and stool culture (HUG)	Positive/Negative
*Entomoeba histolytica*	Stool PCR (HUG)	Positive/Negative
*Trypanosoma cruzi*	Chagas STAT-PAK^®^	Positive/Negative
	ELISA IgG (HUG)	Positive > 1
*Echinococcus multilocularis*	ELISA IgG (UniBern) *E. multilocularis* Em2 *E. multilocularis* Em18	Positive/Negative
*Leishmania* spp.	IFAT	Positive ≥ 80 Negative < 80

ELISA: enzyme-linked immunosorbent assay. SWISS TPH: Swiss Tropical and Public Health Institute. HUG: Hôpitaux Universitaires de Genève. PCR: Polymerase Chain Reaction. UniBern: University Hospital of Bern. IFAT: immunofluorescent antibody test.

**Table 2:** Patient baseline characteristics.

Characteristic	*N* = 406
**Sex, *n* (%)**	
Female	259 (63.7%)
Male	147 (36.3%)
Age, median (range)	42.2 (18–84)
**Nationality, *n* (%)**	
Switzerland/Europe	347 (85.5%)
Others	59 (14.5%)
**Region of origin, *n* (%)**	
Africa	19 (4.7%)
Asia	12 (3.0%)
Mediterranean Basin and Middle East	84 (20.7%)
Latin America	20 (5.0%)
Northern Europe, USA, Australia	271 (66.7%)
**Travel, *n* (%)**	
**Africa**	
for more than 4 consecutive weeks *	30 (7.4%)
less than 4 consecutive weeks or never	317 (78.0%)
unknown duration	59 (14.6%)
**Asia**	
for more than 4 consecutive weeks *	41 (10.0%)
less than 4 weeks or never	284 (70.0%)
unknown duration	81 (20.0%)
**Mediterranean Basin and Middle East**	
for more than 4 consecutive weeks *	111 (27.4%)
less than 4 weeks or never	158 (38.9%)
unknown duration	137 (33.7%)
**Latin America**	
for more than 4 consecutive weeks *	37 (9.1%)
less than 4 consecutive weeks or never	310 (76.3%)
unknown duration	59 (14.5%)
**Disease for which ISIM drugs were prescribed**	
Multiple sclerosis	282 (69.4%)
Rheumatic disease	35 (8.5%)
Dermatological disease	14 (3.4%)
Inflammatory bowel disease	7 (1.7%)
Organ transplantation	17 (4.2%)
Other: including pre transplantation screening	54 (13.3%)
Immunosuppression at the time of the Quantiferon test, *n* (%)	184 (52%) ^◊^
Immunosuppression at the time of the serological screening, *n* (%)	220 (57.6%) ^⌂^

USA: United States of America. ISIM: immunosuppressive and immunomodulatory (ISIM) agents. * Including country of origin. ^◊^ *n* = 353. ^⌂^ *n* = 382.

**Table 3:** Results of screening tests for latent tuberculosis and parasitic infections.

Pathogen	Number of Test Done	Positive *n* (%)	95% CI
*Mycobacterium tuberculosis*	353	24 (6.7%)	4.6–10.0
*Strongyloides* spp.			
• *Strongyloides* spp. in–house ELISA IgG	368	6 (1.6%)	0.7–3.6
• *Strongyloides* spp. ELISA IgG Euroimmune^®^ (Confirmation test)	6	5 (83.3%)	2.3–9.8
• Baermann test and stool culture	38	0	-
• ^a^ Definition of positive: in-house > 0.7 or Euroimmune^®^ positive	368	8 (2.2%)	0.9–4.2
*Entamoeba histolytica*	32	1 (3.1%)	0.1–16
*Trypanosoma cruzi*	64	0	-
*Echinococcus multilocularis*	56	0	-
*Leishmania* spp.	299	1 (0.3%)	<0.1–2.3

^a^ Definition of a positive result has changed 1 September 2018 because a confirmatory test was introduced; patients were considered as positive according to the definition of positive result at the time of the execution of the test. ELISA: enzyme-linked immunosorbent assay.

**Table 4:** Risk factors for latent tuberculosis infection (LTBI) and Strongyloidiasis.

Characteristic	Positive	Negative 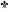	Crude PR (95% CI)	Adjusted PR * (95% CI)
**LTBI**				
**Originate from high prevalence regions**				
No	8 (3.3%)	231	Ref	Ref
Yes	16 (14.0%)	98	**4.2 (1.8–9.5)**	**4.0 (1.8–8.9)**
**Travel**				
No	6 (3.1%)	184	Ref	Ref
Yes	18 (11.0%)	145	**3.5 (1.4–8.6)**	**3.4 (1.4–8.2)**
**Immunosuppression**				
No	15 (8.9%)	154	Ref	Ref
Yes	9 (4.9%)	175	0.5 (0.2–1.2)	
**Age**	-	-	1.0 (0.9–1.0)	1.0 (0.9–1)
**Sex**				
Female	17 (7.5%)	208	Ref	Ref
Male	7 (5.5%)	121	0.7 (0.3–1.7)	0.7 (0.3–1.6)
**Strongyloidiasis**				
**Originate from endemic country**				
No	3 (1.2%)	240	Ref	-
Yes	5 (4.0%)	120	3.2 (0.8–13.4)	-
**Travel**				-
No	3 (1.6%)	183	Ref	-
Yes	5 (2.7%)	177	1.7 (0.4–7.0)	
**Immunosuppression**				
No	7 (3.2%)	211	Ref	-
Yes	1 (0.6%)	149	**0.1 (0.01–0.8)**	-
**Age**	-	-	1.0 (0.9–1.0)	-
**Sex**				
Female	3 (1.3%)	226	Ref	
Male	5 (3.6%)	132	2.8 (0.7–11.5)	-

PR: odd ratio; CI: confidence interval. * Adjusted by immunosuppression status for tuberculosis. 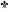 For TB indeterminate results considered as negative.

## Data Availability

The data presented in this study are available on request from the corresponding author. The data are not publicly available due to privacy reason.

## Supplementary Materials

The following are available online at https://www.mdpi.com/article/10.3390/tropicalmed6030170/s1, Table S1: list of immunomodulatory or immunosuppressive treatments in alphabetic order.
