# Supplementary material for: Screening for Parasitic Infection and Tuberculosis in Immunosuppressed and Pre-Immunosuppressed Patients: An Observational Study
Source: Trop Med Infect Dis. 2021 Sep 21;6(3):170. doi: 10.3390/tropicalmed6030170 (PMC8482080; doi:10.3390/tropicalmed6030170)
Supplement: Supplementary file 1 [file tropicalmed-06-00170-s001.zip › tropicalmed-1365882-supplementary.pdf]

Supplementary Materials 1: List of Immunomodulatory or Immunosuppressive Treatment in Alphabetic Order.

abatacept	belimumab	daclizumab	leflunomide	rituximab
adalimumab	basiliximab	dimethyl fumarate	6-mercaptopurine	secukinumab
alemtuzumab	baracitinib	eculizumab	methotrexate	sirolimus,
anakinra	canakinumab	etanercept	mycophenolate	tacrolimus
apremilast	certolizumab	everolimus	ocrelizumab	teriflunomide
atacicept	cyclosporine	golimumab	omalizumab	tocilizumab
azathioprine	cyclophosphamide	infliximab	prednisone-equivalent ≥ 20mg/day	ustekinumab
